# The Impact of Proximal Turn-Up Anastomosis Without Adhesive Repair Using BioGlue for Acute Type A Aortic Dissection

**DOI:** 10.7759/cureus.95380

**Published:** 2025-10-25

**Authors:** Kazuhisa Sakamoto, Takeshi Shimamoto, Makoto Takehara, Atsushi Nagasawa

**Affiliations:** 1 Cardiovascular Surgery, Hamamatsu Rosai Hospital, Hamamatsu, JPN; 2 Cardiovascular Surgery, Kyoto University Hospital, Kyoto, JPN; 3 Cardiovascular Surgery, Tenri Hospital, Tenri, JPN

**Keywords:** acute type a aortic dissection, aortic surgery, bleeding risk, surgical anastomosis, surgical glue

## Abstract

Introduction

The prognosis for surgery on acute type A aortic dissection (ATAAD) is poor because the dissected vessel is fragile and prone to bleeding. We have applied a turn-up anastomosis, in which both the Dacron graft and native aortic wall are everted for secure anastomosis. BioGlue (CryoLife, Inc., Kennesaw, GA, USA) has also been occasionally used as an adjunct. However, several potential complications associated with the use of BioGlue have already been reported. This study aimed to compare the clinical impact of BioGlue in patients undergoing ATAAD surgery using proximal turn-up anastomosis.

Materials and methods

Between November 2020 and March 2024, 78 cases were included for ATAAD. The proximal anastomosis was performed with a turn-up anastomosis in all cases, and BioGlue was used as an adjunct in 49 cases (BG). The cases without BioGlue (NBG: 29) and BG were compared.

Results

There were no differences in age, gender, preoperative shock, cardiac tamponade or malperfusion between the two groups. The replacement of ascending aorta was more common in NBG (52%, p=0.0004), while the frozen elephant trunk technique was more common in BG (59%, p=0.0004). Operation time was similar (NBG: 302 min, BG: 277 min, p=0.1451), and no significant difference in intraoperative bleeding (NBG: 1487 mL, BG: 1370 mL, p=0.6942). Both groups had no cases requiring re-anastomosis or repair under re-aortic cross-clamp. In-hospital mortality was 0% in NBG and 14% in BG (The risk difference: 0.142857 (0.044879-0.240835), p=0.0419). Re-exploration for bleeding was similar (NBG: 10%, BG: 6.1%, OR=0.565 [0.106-3.00], p=0.6651). These results were similar for the replacement of ascending aorta only. Postoperative CT scans showed no difference in complete thrombosis of the sinus of Valsalva (p=0.7019). The mean follow-up was 20 months, with one case of aortic root enlargement in BG.

Conclusions

Proximal turn-up anastomosis without BioGlue was shown to be non-inferior to that using BioGlue. Proximal turn-up anastomosis without BioGlue is considered an acceptable technique. The issue of potential late complications remains, and the use of BioGlue should be approached with caution, with its application appropriately limited.

## Introduction

Acute type A aortic dissection (ATAAD) is recognized as a condition that poses a serious threat to life. The mortality rate associated with conservative treatment exceeds 50%, and thus surgical intervention is mostly selected. Over the past two decades, surgical treatment has evolved considerably, and while there has been a decline in mortality rates, they remain approximately 10%. The management of ATAAD represents a significant challenge in the domain of cardiovascular surgery [1,2]. This life-threatening condition requires immediate surgical intervention and may be further complicated by significant coagulopathy. Over the years, numerous surgical adhesives have been developed [3-7]. Traditionally, these adhesives have been utilized, particularly within the false lumen, to secure anastomoses, achieve immediate hemostasis, and reinforce suture lines. One such adhesive is BioGlue® surgical adhesive (CryoLife, Inc., Kennesaw, GA, USA) (BioGlue). While some clinical outcomes have been promising, the potential complications associated with the use of BioGlue - including issues related to wound healing, pseudoaneurysm formation, embolization, and sterile peri-graft abscesses - are increasingly under scrutiny [8-12]. Each complication is considered a potentially life-threatening issue for the patient. Particularly, the proximal anastomosis plays a key role in ATAAD surgery because complications of the aortic root involving the coronary artery and aortic regurgitation are more serious than distal aorta. Although several reapproximation and anastomosis methods of dissected aorta have been suggested, it is crucial to restore the aortic wall while achieving hemostasis and obliterating the flow to the false lumen in order to avoid life-threatening complications. A previous report indicated that the turn-up anastomosis technique is effective for securing the anastomosis during aortic repair [13]. The basic technique of turn-up anastomosis involves firstly inverting the prosthetic graft using multiple (5-8) circumferential interrupted U sutures, followed by the addition of continuous sutures [14]. It was hypothesized that proximal turn-up anastomosis would enable a secure anastomosis of face-to-face attachment of prosthesis and native aorta to be achieved without the use of BioGlue and avoid potentially fatal complications that can occur at the proximal side. This approach has the potential to prevent complications associated with adhesives during the perioperative phase and in subsequent follow-up periods. Notwithstanding the preliminary findings, the efficacy of this approach has not been fully evaluated. This study aimed to compare the clinical impact of BioGlue in patients undergoing ATAAD surgery using proximal turn-up anastomosis, with perioperative outcomes related to intraoperative bleeding as the primary endpoint.

## Materials and methods

Human ethics and consent to participate

The study was conducted in accordance with the guidelines set out in the Declaration of Helsinki. The present research protocol was approved by the Institutional Review Board of the Medical Ethics Committee of Hamamatsu Rosai Hospital (Reference number: 2024-08) on June 19, 2024. Due to the implementation of retrospective enrolment, the requirement for written informed consent from patients was waived, and the consent of research participants was obtained using an opt-out system.

Study population

Between November 2020 and March 2024, we conducted a total of 83 consecutive emergency operations for ATAAD at our institution. This study encompasses 78 cases, excluding five cases in which either valve-sparing root repair or aortic root replacement utilizing a composite graft was performed. We compared 49 cases in which BioGlue was administered into the false lumen for proximal reinforcement (BG) with 29 cases in which BioGlue was not utilized (NBG).

Operative techniques

**Figure 1:**
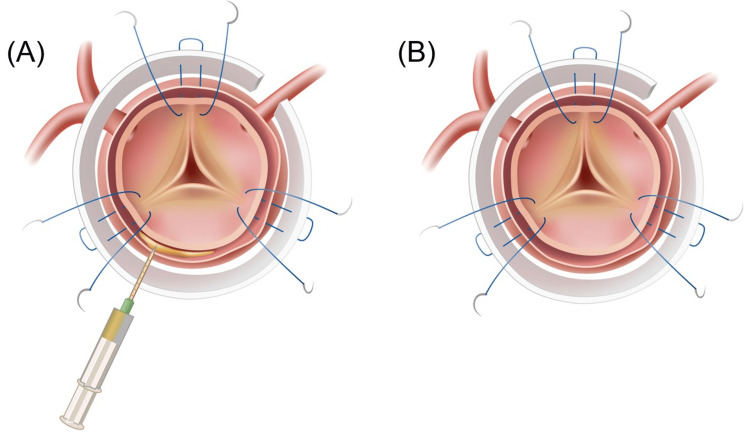
(A) Proximal turn-up anastomosis with BioGlue. Proximal reinforcement was achieved by injecting BioGlue into the false lumen. (B) Proximal turn-up anastomosis without BioGlue. Nothing is injected into the false lumen. Image credit: Created by COLBO Co., Ltd., for the authors.

Cardiopulmonary bypass was established with arterial blood flow from the femoral artery or axillary artery or ascending aorta and venous drainage from the right atrium or bilateral vena cava. The femoral artery was the preferred cannulation site; however, if this was not feasible, alternative sites were utilized. Circulatory arrest was implemented once the bladder or rectal temperature reached 28°C. Subsequently, the ascending aorta was transected, and the orifices of the arch branches were verified, followed by the rapid insertion of a cannula to commence antegrade cerebral perfusion through these orifices. A cardioplegic solution was administered retrogradely through the coronary sinus. Our fundamental approach involved entry resection; if a primary tear was identified in the ascending aorta, we proceeded with the replacement of the ascending aorta (AAR). Conversely, if the tear was located in the aortic arch, we opted for either total or partial aortic arch replacement. The Frozen Elephant Trunk Technique (FET) was actively applied in cases where the entry was located in the descending aorta or where the true lumen was collapsed. The dimensions of the sinotubular junction (STJ) were assessed using a cylinder sizer, which informed the determination of graft size. In cases where a size discrepancy was noted between the distal and proximal anastomosis, particularly during ascending aorta replacement, the graft was meticulously adjusted to ensure precise alignment with the dimensions of the distal anastomosis site. Upon completion of the distal anastomosis, lower body circulation was reinstated through rewarming, followed by the execution of the proximal anastomosis after transecting the ascending aorta 1 cm above the level of the STJ. In BG, BioGlue was injected into the false lumen (Figure 1A), whereas in NBG, no injection or manipulation of the false lumen was conducted (Figure 1B). Throughout the study period, four surgeons performed operations for ATAAD, with the decision to utilize BioGlue not being contingent upon the extent of progression of the dissection of the sinus of Valsalva but rather based on the surgeon's preference. Our basic method of proximal anastomosis was a turn-up anastomosis with six fixed points. All points were placed at the same angle, and three sutures were placed at each commissure to secure the anastomosis. In the case of arch repair, after the aorta was declamped, the arch branches were reconstructed under spontaneous heart beating.

Data collection

All data, including late events, were retrospectively obtained from medical records and the electronic patient record system (EGMAIN-GX, Fujitsu, Tokyo). After surgery, a contrast-enhanced multi-slice CT scan was performed prior to the patient's discharge from the hospital, unless it was deemed impossible. The diameter of the sinus of Valsalva was measured utilizing multiplanar reconstruction techniques using the high-resolution software (Ziostation2 [Ziosoft, Inc., Tokyo, Japan]). The diameter of the sinus of Valsalva was precisely measured perpendicular to the plane of the aortic annulus. It was defined as the distance between the commissure and the corresponding opposite sinus. The measurement was taken by one doctor (KS).

Endpoint

The primary endpoint was operative outcomes and the re-exploration rate due to bleeding. The secondary endpoints included early results, excluding the re-exploration rate due to bleeding; overall survival rates; the incidence of aorta-related death; and the incidence of proximal anastomosis-related events. Additionally, postoperative CT findings were evaluated.

Definition

Stroke was defined as any neurological deficit that is corroborated by imaging studies. Acute renal failure was characterized by a serum creatinine level exceeding 2.0 mg/dL or more than double the preoperative baseline level. Lung complications encompassed the necessity for tracheostomy, the requirement for mechanical ventilation for 24 hours or longer, and/or the development of pneumonia. Overall survival was defined as the duration from the date of surgery until death from any cause. Aorta-related death was specified as mortality attributable to aortic disease, mortality resulting from additional treatment, or sudden death. The incidence of aorta-related death was measured as the time interval from the date of surgery to the occurrence of aorta-related death. Proximal anastomosis-related events were defined as any one or a combination of the following: a 5 mm or greater dilation of the aortic root, re-dissection of the aortic root, exacerbation of aortic valve insufficiency, or the necessity for additional surgical intervention related to the proximal anastomotic lesion for any reason. The incidence of proximal anastomosis-related events was defined as the period from the date of surgery to the occurrence of such events.

Statistical analysis

This study did not include a formal power analysis as it was an observational study. Summary statistics were constructed using frequencies and proportions for categorical data. For continuous variables, the mean and standard deviation were calculated, with the standard deviation presented in parentheses. Fisher's exact test was employed to compare categorical data. Both the odds ratio (OR) and the absolute risk difference were calculated to quantify the effect size in early results. Continuous variables were analyzed using the Student’s t-test when variances were equal, and the Welch’s t-test when variances were unequal. In order to mitigate the impact of surgical procedures, a subgroup analysis was conducted for the primary endpoint of operative outcomes and early results, with the focus being solely on AAR. Freedom from all-cause death was estimated using the Kaplan-Meier method. Comparisons between the two groups were conducted using the log-rank test. Hazard ratios (HR) and 95% confidence intervals (CI) were estimated using the Cox proportional hazards model. For the incidence of aorta-related death, we performed a competing-risk analysis in which death of other than aorta-related cause was considered as a competing risk. The incidence of proximal anastomosis-related events was analyzed similarly, with death from any cause as a competing risk. The cumulative incidences were also compared between the two groups with the use of Gray test. HR and 95% CI were calculated using the Fine and Gray method. All P-values were two-sided, with a p-value of less than 0.05 considered statistically significant. All analyses were performed using JMP Pro 17.2 (SAS Institute, Cary, NC, USA) and EZR (Jichi Medical University, Tochigi, Japan), which is a graphical user interface for R (The R Foundation for Statistical Computing, Vienna, Austria).

## Results

Patient characteristics are shown in Table 1. The mean age was 69 (14) years in NBG and 69 (13) years in BG, with no statistically significant differences observed between the two groups. Furthermore, there were no notable differences in sex distribution, prevalence of hypertension, hyperlipidemia, diabetes mellitus, chronic obstructive pulmonary disease, smoking habits, or history of re-sternotomy. Notably, the general condition of patients immediately prior to surgery was comparable between NBG and BG. Specifically, the incidences of shock, cardiac tamponade, and cerebral, visceral, and limb malperfusion were remarkably similar across both groups. EuroSCORE II predicted mortality was 8.3 (7.0)% in NBG and 10.5 (12.3)% in BG, with no significant difference between the two groups (p=0.3159). Additionally, none of the patients in this cohort were diagnosed with a connective tissue disorder.

**Table 1:** Patient characteristics. SD: standard deviation, HT: hypertension, HL: hyperlipidemia, DM: diabetes mellitus, COPD: chronic obstructive pulmonary disease.

	NBG (N=29)	BG (N=49)	p-value
Age (SD)	69 (14)	69 (13)	0.9823
Sex; male	14 (48%)	32 (65%)	0.1595
HT	24 (83%)	43 (88%)	0.7377
HL	8 (28%)	14 (29%)	1.0
DM	2 (6.9%)	5 (10%)	1.0
COPD	1 (3.5%)	3 (6.1%)	1.0
Smoking habitus	8 (28%)	25 (51%)	0.0584
Shock status	4 (14%)	9 (18%)	0.7571
Cardiac tamponade	4 (14%)	10 (20%)	0.5524
Cerebral malperfusion	2 (6.9%)	8 (16%)	0.3066
Visceral malperfusion	0	2 (4%)	0.5268
Limb malperfusion	4 (14%)	12 (24%)	0.3854
Re-sternotomy	0	1 (2%)	1.0
EuroSCORE II (SD)	8.3 (7.0) %	10.5 (12.3) %	0.3159
Time distribution			0.06002
June 2022 (N=39)	19 (66%)	20 (41%)	
July 2022 (N=39)	10 (34%)	29 (59%)	

Operative outcomes for all cases are presented in Table 2. In both groups, femoral artery cannulation was employed in over half of the cases. AAR was 15 cases in NBG and 6 cases in BG, indicating a higher proportion in NBG (p=0.0004). There were no significant differences between the two groups regarding the replacement of the partial aortic arch and the total aortic arch. Furthermore, FETs were performed more frequently in BG compared to NBG (p=0.0004). The duration of the operation, cardiopulmonary bypass time, cardiac ischemia time, and circulatory arrest time did not differ significantly between the two groups. However, the antegrade cerebral perfusion time was longer in BG than in NBG, which can be attributed to variations in surgical technique. No significant differences were noted in terms of intraoperative bleeding or the amount of blood transfusion required. Additionally, there were no cases in either group that necessitated re-proximal anastomosis or repair under re-aortic cross-clamp. The results of the analysis focusing solely on isolated AAR, conducted to eliminate procedural bias, are detailed in Table 3. When limited to AAR cases, there was no difference in antegrade cerebral perfusion time. Consistent with the overall analysis, the examination of AAR cases revealed no significant differences in intraoperative bleeding or the amount of blood transfusion.

**Table 2:** Operative outcomes (All) SD: standard deviation.

	NBG (N=29)	BG (N=49)	p-value
Cannulation site			
Femoral artery	25 (86%)	33 (67%)	0.1061
Axillary artery	1 (3%)	4 (8%)	0.6457
Ascending aorta	3 (10%)	12 (24%)	0.1492
Range of Replacement			
Ascending aorta	15 (52%)	6 (12%)	0.0004
Partial aortic arch	5 (17%)	5 (10%)	0.4866
Total aortic arch	5 (17%)	9 (18%)	1.0
Total aortic arch + FET	5 (17%)	29 (59%)	0.0004
Operation time, min. (SD)	302 (80)	277 (58)	0.1451
Cardiopulmonary bypass, min. (SD)	156 (47)	157 (29)	0.8916
Cardiac ischemia, min. (SD)	92 (25)	93 (19)	0.7636
Circulatory arrest, min. (SD)	42 (9)	40 (7)	0.3192
Antegrade cerebral perfusion, min (SD)	75 (47)	97 (30)	0.0347
Minimum temperature, ℃ (SD)	26 (1.2)	26 (1.4)	0.3655
Intraoperative bleeding, ml (SD)	1487 (1216)	1370 (1290)	0.6942
Transfusion			
Red blood cell, unit (SD)	8.9 (5.1)	8.5 (5.8)	0.7962
Fresh frozen plasma, unit (SD)	7.9 (4.7)	7.7 (4.1)	0.8168
Platelet concentrate, unit (SD)	23 (7.1)	21 (4.8)	0.2149

**Table 3:** Operative outcomes (The replacement of ascending aorta only). SD: standard deviation.

	NBG (N=15)	BG (N=6)	p-value
Operation time, min. (SD)	255 (53)	302 (120)	0.3891
Cardiopulmonary bypass, min. (SD)	126 (20)	158 (58)	0.2446
Cardiac ischemia, min. (SD)	75 (13)	95 (39)	0.2544
Circulatory arrest, min. (SD)	38 (10)	39 (9)	0.9177
Antegrade cerebral perfusion, min (SD)	35 (23)	33 (6.7)	0.7953
Minimum temperature, ℃ (SD)	26 (1.6)	26 (1.6)	0.4988
Intraoperative bleeding, ml (SD)	1100 (628)	2835 (3214)	0.2499
Transfusion			
Red blood cell, unit (SD)	9.1 (5.5)	14 (5.3)	0.0612
Fresh frozen plasma, unit (SD)	6.5 (4.4)	9.3 (5.0)	0.2179
Platelet concentrate, unit (SD)	23 (7.0)	23 (8.2)	0.8649

Early results for all cases are summarized in Table 4. No in-hospital deaths were observed in NBG; however, seven patients (14%) died prior to discharge in BG. The causes of in-hospital death in BG were multiple organ failure in three cases, acute myocardial infarction and low cardiac output syndrome in one case, bowel ischemia in one case, gastrointestinal bleeding in one case, and pulmonary artery embolism in one case. The in-hospital mortality rate was significantly higher in BG (p=0.04). The risk difference in in-hospital mortality was 0.142857, with a 95% CI ranging from 0.044879 to 0.240835. No significant differences were observed in the rate of re-exploration for bleeding between the two groups (NBG: 3 (10%), BG: 3 (6%), OR=0.565, 95% CI 0.106-3.00, p=0.6551). Although the incidence of atrial fibrillation was greater in BG (OR=3.39, 95% CI 1.18-9.78, p=0.0289), no differences were noted in other postoperative complications. The analysis of AAR is detailed in Table 5. As was the case with the overall analysis, BG showed a higher in-hospital mortality rate compared to NBG (risk difference: 0.5 [95% CI: 0.099924-0.900076], p=0.0150), and the risk difference in re-exploration rate was -0.13333, with a 95% CI ranging from -0.30536 to 0.038694, indicating no statistically significant difference between groups. With regard to the occurrence of atrial fibrillation, no statistically significant discrepancy was identified, contrasting with the outcomes of the overall analysis (OR=8, 95% CI 0.93-66.4, p=0.1196).

**Table 4:** Early results (All) OR: odds ratio, CI: confidence intervals, NA: not applicable

	NBG (N=29)	BG (N=49)	OR (95% CI)	p-value
In-hospital mortality	0	7 (14%)	NA	0.0419
Re-exploration	3 (10%)	3 (6.1%)	0.565 (0.106-3.00)	0.6651
Stroke	6 (20%)	4 (8%)	0.341 (0.087-1.33)	0.1611
Lung complication	9 (31%)	13 (27%)	0.802 (0.292-2.20)	0.7955
Tracheostomy	2 (6.9%)	2 (4.1%)	0.574 (0.076-4.31)	0.6254
Acute renal failure	7 (24%)	21 (44%)	2.44 (0.878-6.81)	0.0939
Deep wound infection	0	1 (2.0%)	NA	1.0
Bowl ischemia	0	2 (4.1%)	NA	0.5268
Atrial fibrillation	6 (20%)	23 (47%)	3.39 (1.18-9.78)	0.0289
Recurrent nerve paralysis	0	1 (2.0%)	NA	1.0
Home discharge	21 (72%)	36 (73%)	1.05 (0.376-2.96)	1.0

**Table 5:** Early results (The replacement of ascending aorta only) OR: odds ratio, CI: confidence intervals, NA: not applicable

	NBG (N=15)	BG (N=6)	OR (95% CI)	p-value
In-hospital mortality	0	3 (50%)	NA	0.0419
Re-exploration	2 (13%)	0	NA	0.6651
Stroke	4 (27%)	1 (17%)	0.55 (0.048-6.27)	0.1611
Lung complication	4 (27%)	3 (50%)	2.75 (0.385-19.7)	0.7955
Tracheostomy	1 (6.7%)	0	NA	0.6254
Acute renal failure	4 (27%)	3 (50%)	2.75 (0.385-19.7)	0.0939
Deep wound infection	0	1 (17%)	NA	1.0
Bowl ischemia	0	1 (17%)	NA	0.5268
Atrial fibrillation	3 (20%)	4 (67%)	8 (0.93-66.4)	0.0289
Recurrent nerve paralysis	0	0	NA	1.0
Home discharge	11 (73%)	2 (33%)	0.182 (0.023-1.41)	1.0

**Figure 2:**
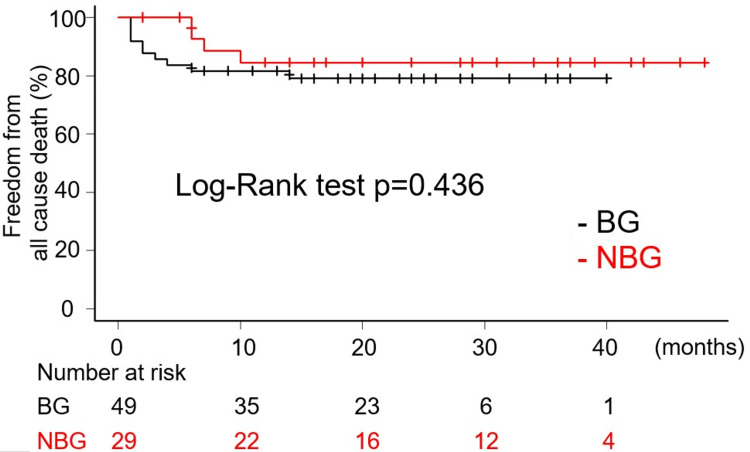
Kaplan-Meier curve showing freedom from all-cause death.

**Figure 3:**
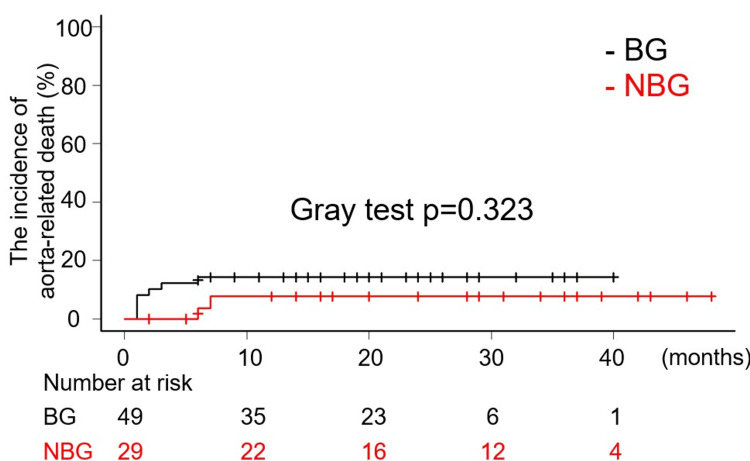
Kaplan-Meier curve showing freedom from aorta-related death.

**Figure 4:**
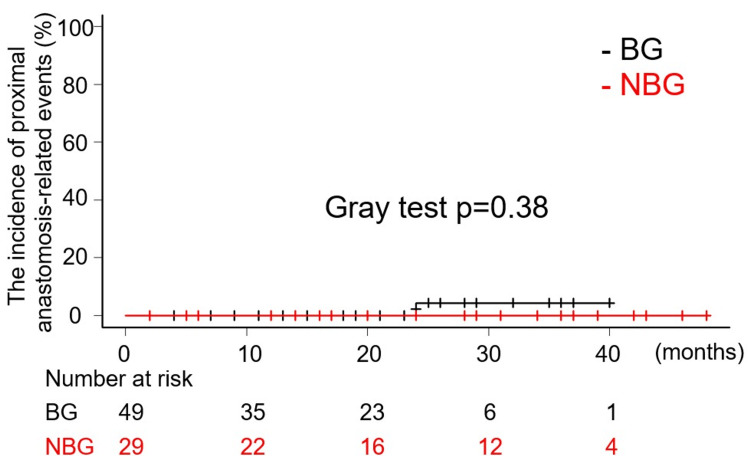
Kaplan-Meier curve showing freedom from the incidence of proximal anastomosis-related events.

The mean and median follow-up period were 20 (12) and 19.5 (interquartile range of 8.5 to 29) months. A total of five late deaths were recorded, comprising two in BG and three in NBG. In BG, one patient succumbed to pneumonia, while the other died from frailty associated with advanced age. In NBG, the causes of death included heart failure, pneumonia, and frailty due to old age. As demonstrated in Figure 2, the Kaplan-Meier curves illustrate the overall survival rates. There was no statistically significant difference in the freedom from all-cause death rates between NBG and BG (Log-Rank test p=0.436, HR: 0.6361, 95% CI: 0.1995-2.029). Notably, there were no aorta-related deaths reported in either group following discharge, and no significant differences were observed in the incidence of aorta-related death between the two groups (Gray test p=0.323, HR: 0.4671, 95% CI: 0.1014-2.152) (Figure 3). One late event related to the proximal anastomosis was documented in BG. During the initial operation, it was determined that the dissection extended through all the sinuses of Valsalva, with the primary tear located in the STJ, slightly peripheral to the left coronary artery orifice. This entry was resected, and a proximal anastomosis was subsequently performed using the previously described technique. The patient experienced an uneventful postoperative course and was discharged from the hospital. However, 24 months after the initial procedure, the patient required aortic root replacement due to dilatation of the aortic root and worsening aortic valve insufficiency. Notably, the intima surrounding the orifice of the right coronary artery was thinned and had disappeared, leaving only the adventitia. Additionally, the aortic wall and the surrounding tissue around both coronary orifices exhibited stiffness and inflammatory thickening. Due to significant periaortic adhesions, a valve-sparing root replacement could not be performed. In contrast, no late events related to proximal anastomosis were observed in NBG. Figure 4 demonstrates the cumulative incidence curve of events related to proximal anastomosis. (Gray test p=0.38, HR: 0.00001781, 95% CI: 0.000002495 - 0.00001271). Further interventions on the downstream aorta during the follow-up periods were documented in one case in NBG and three cases in BG. It was observed that all cases were thoracic endovascular aortic repair (TEVAR).

Evaluation of the sinus of Valsalva utilizing computed tomography scan

The evaluation of the sinus of Valsalva is presented in Table 6. All patients underwent a preoperative CT scan. The preoperative diameter of the sinus of Valsalva was measured at 38 (4.9) mm in NBG and 39.8 (5.1) mm in BG, with no statistically significant difference observed between the two groups (p=0.1313). Postoperative evaluation via CT scan was conducted for 29 patients in NBG and 47 patients in BG. Postoperative CT scans were not possible for the two cases in BG, as they both deceased early following their surgical procedures. The diameter of the sinus of Valsalva following surgery was recorded as 35.7 (3.9) mm in NBG and 36.6 (4.6) mm in BG, with no significant difference noted (p=0.3673). In NBG, complete thrombosis of the sinus of Valsalva was achieved in 27 cases (93.1%), while in BG, this was achieved in 42 cases (89.4%). No significant difference was found between the two groups (p=0.7019). During the follow-up period, there were no cases of aortic root enlargement of 5 mm or more in NBG; however, one case was reported in BG.

**Table 6:** Evaluation of the sinus of Valsalva using CT scan. SD: standard deviation, NA: not applicable. SV: the sinus of Valsalva

	NBG (N=29)	BG (N=49)	p-value
Preoperative diameter, mm (SD)	38 (4.9)	39.8 (5.1)	0.1313
Postoperative evaluation	29 (100%)	47 (96%)	NA
Postoperative diameter, mm (SD)	35.7 (3.9)	36.6 (4.6)	0.3673
Complete thrombosis of SV	27 (93.1%)	42 (89.4%)	0.7019

## Discussion

This study assessed the clinical impact of BioGlue in the context of turn-up anastomosis for proximal anastomosis during aortic arch repair in patients with ATAAD. The hypothesis posited that the application of BioGlue would lead to a significant reduction in anastomotic bleeding and related hemorrhagic events. Consequently, various parameters related to bleeding and hemostasis were examined, including intraoperative blood loss, volume of blood transfusion, duration of the operation, rates of re-exploration for postoperative bleeding, and associated mortality rates. To mitigate bias associated with the extent of surgical repair - which can markedly affect the range of aortic replacement - and the surgical techniques employed, subgroup analyses were confined to cases of AAR. The results indicated that the use of BioGlue did not lead to a significant decrease in intraoperative bleeding or operative duration when compared to cases where BioGlue was not utilized. This finding was consistent even when analyses were limited to AAR cases. Furthermore, no significant differences were noted in the rates of re-exploration due to bleeding or in the incidence of postoperative complications, with the exception of a higher incidence of atrial fibrillation observed in BG. However, the higher incidence of atrial fibrillation in BG patients was no longer significant in the subgroup analysis, suggesting that this was due to the influence of surgical technique selection rather than a relationship with BioGlue. Importantly, the in-hospital mortality rate was significantly higher in BG (14%) compared to NBG (0%). A thorough analysis indicated that other factors, including patient demographics and preoperative conditions, did not differ significantly between the two groups. However, given that the procedure is more frequently performed in BG with more invasive FETs, it remains inconclusive whether the increased in-hospital mortality is directly attributable to the use of BioGlue. It is conceivable that the observed in-hospital mortality may be influenced by factors unrelated to the application of BioGlue. Nonetheless, the findings suggest that the use of BioGlue did not contribute to a reduction in in-hospital mortality rates.

The findings of this study do not suggest that BioGlue is universally ineffective. Introduced in the late 1990s and early 2000s, BioGlue has gained prominence in vascular and cardiac surgery, particularly for achieving hemostasis at anastomotic sites that are susceptible to significant bleeding, such as those encountered in aortic surgery. In 2001, BioGlue received approval from the Food and Drug Administration (FDA) for use in the United States, resulting in a substantial increase in its application in cardiovascular surgery, particularly in aortic repair procedures. Its utilization has been extensively expanded to include minimally invasive and anatomically complex surgeries where effective hemostasis is critical. The efficacy of BioGlue in these contexts has been well-documented in the literature [15-17]. However, several reports have emerged that highlight the adverse effects associated with the use of BioGlue. Notably, excessive tissue reactions and tissue damage have been reported, leading to tissue sclerosis and foreign body reactions [18-20]. Concerns have been raised regarding the safety of BioGlue in specific surgical scenarios, particularly in instances of over-application or when the adhesive is applied in proximity to sensitive anatomical structures. Inflammatory thickening resulting from excessive foreign body reactions and necrosis of the intima due to its histotoxic properties have been observed during re-operation.

We employ a distinctive technique known as the proximal turn-up anastomosis, utilizing 5-8 pairs of U sutures [13,14]. The fundamental approach to this anastomosis involves the eversion of the prosthetic graft, followed by the application of continuous sutures, complemented by circumferential felt strips. This methodology aims to achieve a face-to-face anastomosis between the dissected aortic wall and the prosthetic graft. As illustrated in Figure 1B, three of the six U sutures are strategically positioned near the apex of the three commissures, which are robust and reliable tissues that facilitate the anastomosis and ensure the successful suturing and fixation of the STJ. Despite the necessity for meticulous attention to the location of the coronary artery orifice when applying this method, it is indeed feasible to create the suture line at the level of the STJ with simultaneous resection of excess dissected aortic wall. This method is highly effective as the felt strips are applied solely to the outer circumference of the anastomosis, thereby avoiding luminal narrowing, and there have been no reports of hemolysis. Nevertheless, despite these advantages of proximal turn-up anastomosis, the prevention of microscopic suture leakage remains a challenge due to the fragility of the dissected intima. To resolve this problem, BioGlue has been applied to reinforce suture lines and prevent potential microscopic suture leakage. However, the results of this study suggest that the benefits of adding BioGlue to surgical procedures have not been proven, and its efficacy remains unproven.

There exists an ongoing debate concerning the necessity of adhesives for achieving secure anastomosis in ATAAD. Some clinicians contend that the use of adhesives is superfluous for ensuring secure anastomosis within the false lumen, citing the potential for increased complications [21, 22]. However, the tissue characteristics of the dissected aorta may be exceptionally fragile, potentially influenced by various factors, including patient race and gender. A previous report indicated that the application of BioGlue in thoracic aortic surgery did not correlate with a heightened incidence of anastomotic pseudoaneurysm formation. Nonetheless, its use was strictly regulated, permitted only in cases of acute aortic dissection or significant tissue fragility [23]. We advocate for the selective application of BioGlue, emphasizing that its use should be customized to the specific conditions of individual patients and the particular surgical context. We assert that the utilization of BioGlue necessitates rigorous long-term follow-up to monitor for potential delayed complications. In one observed case involving BioGlue, a patient required reoperation due to aortic root enlargement (52 mm) as determined by CT scan, alongside a progression of moderate aortic valve insufficiency and clinically significant left ventricular dilatation, as assessed through transthoracic echocardiography. Currently, there are no established guidelines for the outpatient follow-up of patients following aortic dissection surgery, although many centers routinely conduct CT scans. We recommend that echocardiography be incorporated into follow-up protocols when deemed necessary to mitigate the risk of significant adverse events.

This study presents several limitations. Firstly, and most significantly, the research was conducted at a single institution with a relatively small patient sample. Secondly, the application of BioGlue was contingent upon the preferences of the surgeons involved. Lastly, the mean follow-up period was only 20 months. It is plausible that a longer follow-up duration could have yielded different results regarding long-term outcomes. It is evident that further studies are required to evaluate the durability, late complications, and cost-effectiveness of the problems. Such studies should include larger multicenter registries incorporating randomized trials and longer-term follow-up. Furthermore, it is proposed that the histopathological changes associated with BioGlue in reoperations be investigated in order to enhance the biological mechanisms involved in the degeneration of complications.

## Conclusions

In ATAAD surgery with proximal turn-up anastomosis, the utilization of BioGlue did not result in the enhancement of perioperative outcomes, including the reduction of intraoperative bleeding and the necessity for blood transfusions. Although the objective was to achieve proximal reinforcement, the use of BioGlue resulted in equivalent rates of complete thrombosis in the false lumen of the sinus of Valsalva, as determined by CT findings. Notably, one patient in BG required reoperation due to the enlargement of the aortic root. While it is essential to verify long-term outcomes, proximal turn-up anastomosis without BioGlue was shown to be non-inferior to the technique using BioGlue. Proximal turn-up anastomosis without BioGlue is considered an acceptable technique. The issue of potential late complications remains, and the use of BioGlue should be approached with caution, with its application appropriately limited.

## References

[1] Evangelista A, Isselbacher EM, Bossone E (2018). Insights from the International Registry of Acute Aortic Dissection: a 20-year experience of collaborative clinical research. Circulation.

[2] Yoshimura N, Sato Y, Takeuchi H (2025). Thoracic and cardiovascular surgeries in Japan during 2022: annual report by the Japanese Association for Thoracic Surgery. Gen Thorac Cardiovasc Surg.

[3] Guilmet D, Bachet J, Goudot B, Laurian C, Gigou G, Bical O, Barbagelatta M (1979). Use of biological glue in acute aortic dissection: preliminary clinical results with a new surgical technique. J Thorac Cardiovasc Surg.

[4] Bachet J, Goudot B, Dreyfus GD, Brodaty D, Dubois C, Delentdecker P, Guilmet D (1999). Surgery for acute type A aortic dissection: the Hopital Foch experience (1977-1998). Ann Thorac Surg.

[5] Borst HG, Haverich A, Walterbusch G, Maatz W, Messmer B (1982). Fibrin adhesive: an important hemostatic adjunct in cardiovascular operations. J Thorac Cardiovasc Surg.

[6] Morita S, Matsuda T, Tashiro T, Komiya T, Ogino H, Mukohara N, Tominaga R (2020). Randomized clinical trial of an elastomeric sealant for hemostasis in thoracic aortic surgery. Gen Thorac Cardiovasc Surg.

[7] Morita S, Yaku H (2023). A sealant with a hemostatic mechanism independent of the blood coagulation function was effective in both elective and emergency surgery for thoracic aorta. Gen Thorac Cardiovasc Surg.

[8] Pasic M, Unbehaun A, Drews T, Hetzer R (2011). Late wound healing problems after use of BioGlue for apical hemostasis during transapical aortic valve implantation. Interact Cardiovasc Thorac Surg.

[9] Kobayashi T, Kurazumi H, Sato M, Gohra H (2018). Pseudoaneurysm rupture after acute Type A dissection repair: possible reaction to BioGlue. Interact Cardiovasc Thorac Surg.

[10] Miyagi T, Ishimine T, Nakazato J (2021). Coronary artery embolism caused by BioGlue surgical adhesive after type A acute aortic dissection repair. JACC Case Rep.

[11] Yamasaki M, Abe K, Nakamura R, Tamaki R, Misumi H (2022). BioGlue cerebral embolism following acute type A aortic dissection repair. J Cardiol Cases.

[12] Yilmaz N, Sologashvili T, Huber C, Cikirikcioglu M (2021). Sterile peri-graft abscess formation following aortic replacement: a word of caution for usage of BioGlue(®). Int J Artif Organs.

[13] Tamura N, Komiya T, Sakaguchi G, Kobayashi T (2007). 'Turn-up' anastomotic technique for acute aortic dissection. Eur J Cardiothorac Surg.

[14] Shimamoto T, Komiya T, Matsuo T (2023). Clinical impact of turn-up anastomosis in the treatment of type A acute aortic dissection. Asian Cardiovasc Thorac Ann.

[15] Coselli JS, Bavaria JE, Fehrenbacher J, Stowe CL, Macheers SK, Gundry SR (2003). Prospective randomized study of a protein-based tissue adhesive used as a hemostatic and structural adjunct in cardiac and vascular anastomotic repair procedures. J Am Coll Surg.

[16] Raanani E, Latter DA, Errett LE, Bonneau DB, Leclerc Y, Salasidis GC (2001). Use of “BioGlue” in aortic surgical repair. Ann Thorac Surg.

[17] Passage J, Jalali H, Tam RKW, Harrocks S, O’Brien MF (2002). BioGlue surgical adhesive--an appraisal of its indications in cardiac surgery. Ann Thorac Surg.

[18] LeMaire SA, Schmittling ZC, Coselli JS, Ündar A, Deady BA, Clubb FJ Jr, Fraser CD Jr (2002). BioGlue surgical adhesive impairs aortic growth and causes anastomotic strictures. Ann Thorac Surg.

[19] Fürst W, Banerjee A (2005). Release of glutaraldehyde from an albumin-glutaraldehyde tissue adhesive causes significant in vitro and in vivo toxicity. Ann Thorac Surg.

[20] Slezak P, Klang A, Ferguson J (2020). Tissue reactions to polyethylene glycol and glutaraldehyde-based surgical sealants in a rabbit aorta model. J Biomater Appl.

[21] Dagenais F (2019). To glue or not to glue in type A dissection repair?. J Thorac Cardiovasc Surg.

[22] Yang B (2024). Commentary: It is okay to leave the dissected aortic root alone, but no felt or bio-glue please!. Semin Thorac Cardiovasc Surg.

[23] Ma WG, Ziganshin BA, Guo CF, Zafar MA, Sieller RS, Tranquilli M, Elefteriades JA (2017). Does BioGlue contribute to anastomotic pseudoaneurysm after thoracic aortic surgery?. J Thorac Dis.

